# Discovery, linkage disequilibrium and association analyses of polymorphisms of the immune complement inhibitor, decay-accelerating factor gene (DAF/CD55) in type 1 diabetes

**DOI:** 10.1186/1471-2156-7-22

**Published:** 2006-04-20

**Authors:** Hidenori Taniguchi, Christopher E Lowe, Jason D Cooper, Deborah J Smyth, Rebecca Bailey, Sarah Nutland, Barry C Healy, Alex C Lam, Oliver Burren, Neil M Walker, Luc J Smink, Linda S Wicker, John A Todd

**Affiliations:** 1 Juvenile Diabetes Research Foundation/Wellcome Trust Diabetes and Inflammation Laboratory, Cambridge Institute for Medical Research, University of Cambridge, Hills Road, Cambridge, CB2 2XY, UK

## Abstract

**Background:**

Type 1 diabetes (T1D) is a common autoimmune disease resulting from T-cell mediated destruction of pancreatic beta cells. Decay accelerating factor (DAF, CD55), a glycosylphosphatidylinositol-anchored membrane protein, is a candidate for autoimmune disease susceptibility based on its role in restricting complement activation and evidence that DAF expression modulates the phenotype of mice models for autoimmune disease. In this study, we adopt a linkage disequilibrium (LD) mapping approach to test for an association between the DAF gene and T1D.

**Results:**

Initially, we used HapMap II genotype data to examine LD across the *DAF* region. Additional resequencing was required, identifying 16 novel polymorphisms. Combining both datasets, a LD mapping approach was adopted to test for association with T1D. Seven tag SNPs were selected and genotyped in case-control (3,523 cases and 3,817 controls) and family (725 families) collections.

**Conclusion:**

We obtained no evidence of association between T1D and the *DAF *region in two independent collections. In addition, we assessed the impact of using only HapMap II genotypes for the selection of tag SNPs and, based on this study, found that HapMap II genotypes may require additional SNP discovery for comprehensive LD mapping of some genes in common disease.

## Background

T1D is characterised as a common autoimmune disease, mainly resulting from a T-cell mediated destruction of pancreatic beta cells that leaves patients completely dependent on exogenous insulin to regulate their blood glucose level. T1D is strongly clustered in families with an overall genetic risk ratio, an estimate of the familial clustering of the disease, of approximately 15[1]. However, of the hundreds of association studies reported to date, only four loci have been identified and successfully replicated: the HLA class II genes on chromosome 6p21[2]; the insulin gene (*INS*) on chromosome 11p15[3,4]; *CTLA4 *on chromosome 2q33[5,6]; and *PTPN22 *on chromosome 1p13[7,8]. *CD25 *on chromosome 10p15 has been implicated, but this finding awaits independent replication[9]. Given that these genes alone cannot explain the familial clustering of T1D, many other genes remain to be identified.

Recently, there have been several reports focusing on the relationship between autoimmune disease and the complement system, which is composed of more than 30 soluble and membrane-bound proteins[10,11] and plays an important role in innate host defence. As inappropriate regulation of the complement system can lead to significant damage of host tissues[12], a number of membrane-bound complement regulatory proteins are active, such as DAF, a glycosylphosphatidylinositol-anchored membrane protein that restricts complement activation by inhibiting the formation of C3 convertases in both the classical and alternative pathways[13,14].

Dysfunction of human DAF on erythrocytes contributes to the paroxysmal nocturnal hemoglobinuria (PNH) by increasing their sensitivity to complement lysis[13,15,16]. In addition, a proportion of DAF-deficient (Cromer INAB) patients develop inflammatory bowel disease. However, little is known about DAFs role in autoimmune disease *in vivo*[17].

Recently, it has been reported that DAF modulates T cell immunity by controlling T cell- and antigen-presenting cell- induced alternative pathway of C3 activation during cognate interactions [18-20]. According to gene targeting studies, mice deficient in the DAF1 gene, the murine homologue of human *DAF*, showed more susceptibility to complement mediated inflammatory injury, especially DAF1 deficient female mice in a MRL/lpr background, a model for human systemic lupus erythematosus, which showed aggravated lymphadenopathy and splenomegaly, higher serum anti-chromatin autoantibody levels, and dermatitis[21].

Given this prior evidence, DAF may function as a negative regulator of autoimmune response by modulating T cell activity and directly protecting host tissues *in vivo *and that recombinant DAF may be an ideal therapeutic agent for autoimmunity[22]. On the other hand, *DAF *does not lie under any of the reported T1D linkage peaks[23,24] nor have there been any reports of genetic association studies between *DAF *and autoimmune disease, although recently differential expression of DAF was observed when comparing T cells from nonobese diabetic (NOD) mice and diabetes-resistant NOD mice having a congenic interval containing the DAF gene thereby making it a candidate gene for the *Idd5.4 *region (William Ridgway and Linda Wicker, unpublished observations).

In this study, to elucidate the susceptibility of *DAF *with T1D, we performed an association study using a LD mapping approach, together with the direct analysis of three non-synonymous SNPs (nsSNPs) in large case-control and family collections.

## Results

### Linkage disequilibrium analysis

**Figure 1:**
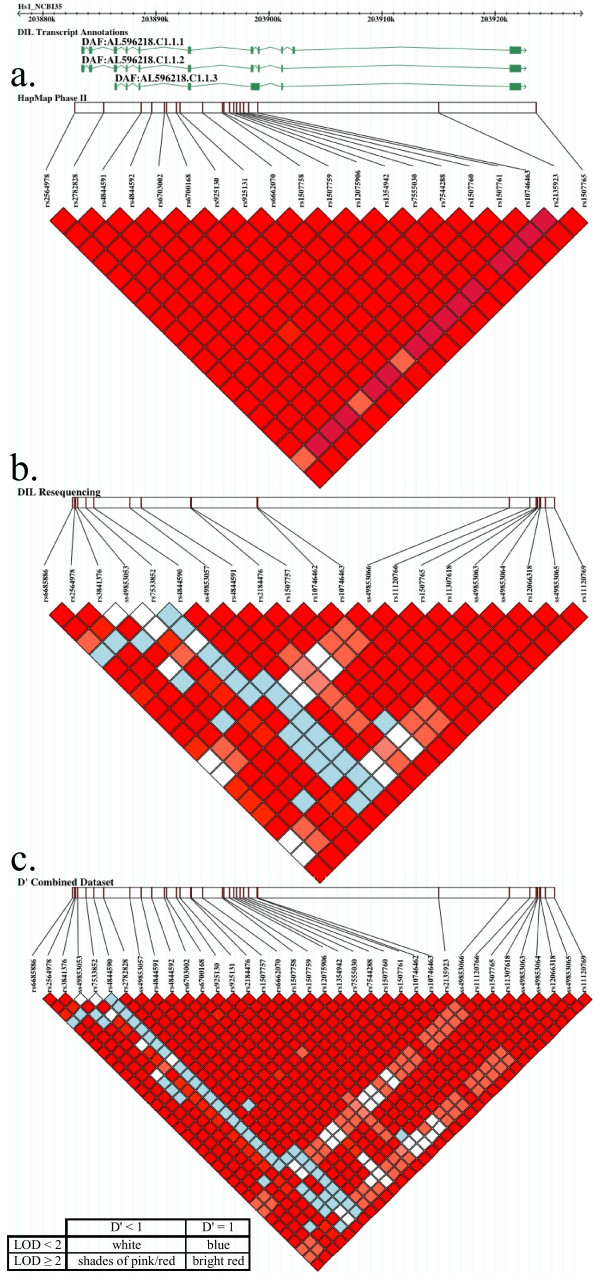
**LD map for human *DAF *region on Chromosome 1q32**. All markers with the MAF of less than 0.05 or with insufficient genotyping data were excluded in the LD measurement. **a. **LD map with 21 markers genotyped in 60 individuals obtained from HapMap II. **b. **LD map with 22 markers identified by resequencing with 32 CEPH's individuals. figure c. LD map with 38 markers, a combined dataset of both HapMap II and in-house resequencing data with 32 CEPH's individuals.

Initially, we used phase II genotyping data from the HapMap project[25,26], a catalogue of common human genetic variants, providing their allele frequencies and intermarker LD patterns among people, within and among populations from African, Asian, and European ancestry. In the *DAF *region, about 40 kb on chromosome 1q32, 21 common SNPs (minor allele frequency (MAF) ≥ 0.05), have been genotyped in 60 U.S.A. residents with northern and western European ancestry, collected in 1980 by the Centre d'Etude du Polymorphisme Humain (CEPH, CEU). We note that all of these SNPs were located in non-coding regions and that the average inter-SNP distance was 2 kb. A LD map of the region, using pairwise D', shows little evidence of recombination within the region (Figure 1a).

### *DAF *resequencing

As we were concerned about adopting a LD mapping approach given the HapMap SNP density[27], we resequenced *DAF *in 32 CEPH individuals, selected from the 60 CEPH individuals used by the HapMap project, to increase the SNP density across the region.

Analysis of the resequencing data identified 32 polymorphisms, 26 of which were SNPs and six were deletion/insertion polymorphisms (DIPs), of which 12 SNPs and four DIPs were novel when compared to dbSNP build 125 (Table 1). Twenty-two polymorphisms were common (MAF ≥ 0.05), five of which were also found in the HapMap II data. The relatively small number of common polymorphisms found in both datasets is not unexpected, as HapMap II SNPs were selected to provide an even coverage in terms of distance across the genome, whereas the resequencing is focused on regions of interest and extracts all common polymorphisms present in these individuals. A LD map of the region, based on these 22 polymorphisms (Figure 1b), revealed additional evidence of recombination within the region over and above that apparent in HapMap II data alone (Figure 1a). There was a breakdown in LD towards the 5' end of *DAF *that was not evident in HapMap II data.

**Table 1:** Polymorphisms identified in human *DAF*. Map positions on human chromosome 1 were from NCBI build 35. Selected tag SNPs are in boldface. Alleles are coded Major > Minor. MAF calculated from the 32 individuals used for tag SNP selection, except for nsSNPs, in which case the MAF is calculated from genotyped controls. DIL = identified by in-house resequencing, HapMap = identified in HapMap II dataset, DIL&HapMap II = Identified in both in-house resequencing and HapMap II dataset.

**Polymorphism – dbSNP(build125)**	**Location**		**MAF**	**Allelic *R***^2^	**Dataset**
				
	**Position(NCBI35)**	**Gene**			
ss49853051(G > C)	203881344	promoter	0.03	-	DIL
ss49853052(C > T)	203881734	promoter	0.03	-	DIL
rs6685886(C > A)	203882613	promoter	0.46	85.07	DIL
**rs2564978(C > T)**	**203882811**	**promoter**	**0.33**	**tag SNP**	**DIL & HapMap**
rs3841376 (TAGTTACTTCCCCTCCTTCCC > -)	203882879	promoter	0.33	100.00	DIL
**ss49853053(A > G)**	**203883074**	**promoter**	**0.26**	**tag SNP**	**DIL**
ss49853054(T > C)	203883723	intron 1	0.02	-	DIL
rs7533852(G > T)	203883822	intron 1	0.44	89.15	DIL
ss49853055(TA > -)	203884424	intron 2	0.03	-	DIL
**rs4844590(C > T)**	**203884524**	**intron 2**	**0.23**	**tag SNP**	**DIL**
rs2782828(G > T)	203885394	intron 2	0.24	94.47	HapMap
ss49853056(A > G)	203886206	intron 2	0.03	-	DIL
**ss49853057(C > G)**	**203887670**	**intron 4**	**0.13**	**tag SNP**	**DIL**
ss49853058(G > A)	203888427	intron 4	0.03	-	DIL
rs4844591(T > C)	203888688	intron 5	0.33	90.27	DIL & HapMap
rs4844592(A > T)	203889606	intron 5	0.33	90.27	HapMap
rs6703002(G > T)	203890767	intron 5	0.32	88.10	HapMap
rs6700168(C > A)	203890929	intron 5	0.38	82.68	HapMap
rs925130(A > G)	203891827	intron 5	0.33	100.00	HapMap
rs925131(G > A)	203892116	intron 5	0.33	90.27	HapMap
ss49853059(C > T)	203892582	intron 5	0.02	-	DIL
**rs2184476(A > G)**	**203893064**	**intron 6**	**0.45**	**tag SNP**	**DIL**
**rs1507757(A > C)**	**203893143**	**intron 6**	**0.35**	**tag SNP**	**DIL**
rs6662070(G > A)	203894104	intron 6	0.31	100.00	HapMap
rs1507758(C > G)	203895876	intron 6	0.33	90.27	HapMap
rs1507759(G > A)	203896023	intron 6	0.32	100.00	HapMap
rs12075906(A > G)	203896494	intron 6	0.33	100.00	HapMap
rs1354942(A > G)	203896887	intron 6	0.33	90.27	HapMap
rs7555030(G > A)	203897125	intron 6	0.33	100.00	HapMap
rs7544288(G > A)	203897417	intron 6	0.41	97.09	HapMap
rs1507760(T > C)	203897760	intron 6	0.33	100.00	HapMap
rs1507761(C > T)	203898194	intron 6	0.33	100.00	HapMap
ss49853060(C > T)	203898910	intron 7	0.04	-	DIL
rs10746462(G > A)	203898943	intron 7	0.33	90.27	DIL
rs10746463(A > G)	203898991	intron 7	0.33	90.27	DIL & HapMap
ss49853061(G > A)	203899255	intron 8	0.02	-	DIL
rs2135923(G > A)	203914988	intron 10	0.33	90.27	HapMap
ss49853066(- > TT)	203921271	intron 10	0.39	97.09	DIL
ss49853062(A > G)	203922987	3'	0.02	-	DIL
rs11120766(A > G)	203923074	3'	0.45	84.94	DIL
rs1507765(A > C)	203923641	3'	0.45	84.94	DIL & HapMap
rs11307618(A > -)	203923723	3'	0.41	97.09	DIL
ss49853063(A > G)	203923730	3'	0.41	97.09	DIL
ss49853064(C > -)	203923933	3'	0.39	91.71	DIL
**rs12066318(T > C)**	**203924026**	**3'**	**0.33**	**tag SNP**	**DIL**
ss49853065(- > ATG)	203924418	3'	0.45	84.94	DIL
rs11120769(G > T)	203925240	3'	0.45	83.28	DIL
rs11117564(C > A)	203925366	3'	0.45	90.45	DIL & HapMap

**nsSNPs**					

rs28371588(>)	203884176	Exon 2	0.05	-	
DAF-WESa/b(T > G)	203884266	Exon 2	0.0055	-	
rs12135160(>)	203899090	Exon 8	-	-	

### Tag SNP analysis of *DAF*

To test for an association between T1D and the *DAF *region, we adopted a LD mapping approach, which exploits the non-random relationships between SNPs (known as LD) in a region of interest to reduce the amount of genotyping required. As the causal SNP is unknown, we assume that predicting the causal SNP is likely to be no more difficult than predicting any other SNP. The predictive performance of the tag SNPs was assessed using a *R*^2 ^measure, which measures the ability to predict each known SNP by multiple regression on the set of tag SNPs. The tag SNPs were analysed using a multilocus test, as described by Chapman *et al*[28], which tests for an association between the tag SNPs and T1D due to LD with one or more causal variants[28,29].

We first combined our resequencing data with the HapMap data, providing a panel of 38 common polymorphisms genotyped in 32 individuals (Table 1), and generated a combined LD map of the region (Figure 1c). Subsequently, seven tag SNPs were selected[9] from the 38 common polymorphisms, required to capture the variation within the *DAF *region with a minimum *R*^2 ^of 0.80[28] (Table 1). The tag SNPs were genotyped in 3,523 cases and 3,817 controls, and in 725 Caucasian multiplex T1D families (Table 2). The case-control and family multilocus *P*-values were 0.12 (3,523 case and 3,817 control genotypes; F_7,7321 _= 1.63) and 0.69 (parent-child trio genotypes = 1,390; χ_7_^2 ^= 4.72), respectively, providing no evidence for the association between T1D and the *DAF *region. In the case-control collection, the multilocus test was stratified by broad geographical within the UK in order to minimize any confounding due to variation in allele frequencies across Great Britain[9,30].

**Table 2:** Genotyping data of tag SNPs. The number of individuals with each genotype in case-control data and the number of alleles in family data are indicated. T, Transmitted. UT, Untransmitted. MAF, minor allele frequency calculated from either control samples or parents.

	Case-Control					Family				
SNP	Population	MAF	1/1	1/2	2/2	Population	MAF	Number of Trios	T	UT

rs2564978(C > T)	Case	0.31	1648	1468	318	UK	0.32	738	483	458
	Control	0.31	1791	1622	354	USA	0.27	526	272	289
ss49853053(A > G)	Case	0.25	1895	1305	219	UK	0.24	736	345	350
	Control	0.24	2147	1338	231	USA	0.29	536	317	304
rs4844590(C > T)	Case	0.23	1987	1194	193	UK	0.21	711	287	309
	Control	0.22	2288	1251	195	USA	0.26	517	272	270
ss49853057(C > G)	Case	0.07	2914	465	22	UK	0.08	709	113	101
	Control	0.08	3071	580	17	USA	0.06	527	62	62
rs2184476(A > G)	Case	0.31	1607	1447	314	UK	0.33	723	475	442
	Control	0.31	1757	1578	345	USA	0.27	492	249	268
rs1507757(A > C)	Case	0.31	1660	1469	319	UK	0.33	736	484	479
	Control	0.31	1756	1586	349	USA	0.27	476	246	248
rs12066318(A > G)	Case	0.44	658	1679	1104	UK	0.43	751	866	859
	Control	0.45	738	1868	1135	USA	0.44	538	597	605

### Analysis of DAF non-synonymous SNPs

Recently, it has been proposed that complex diseases such as T1D may result from the effects of a large number of rare variants, with substantial allelic heterogeneity at causal loci[31,32]. In *DAF*, several rare non-synonymous SNPs (nsSNPs) were reported in the exons encoding the short consensus repeat (SCR) domains of the DAF protein, which have subsequently been shown to be related with antigen of the Cromer blood group system[13,14,33,34]. On the basis of the rare variant hypothesis, we genotyped three rare nsSNPs in3,490 cases and 3,814 controls (Table 3), under the hypothesis that a rare functional variant in *DAF *might have a strong effect in T1D. The following three nsSNPs were assessed: DAF-WES^a/b^(G > T) located in exon 2 with a MAF of 0.0055–0.0060 in a Finnish population[35,36]; rs28371588(C > A), also located in exon 2[34]; and, rs12135160(G > A), identified by SsahaSNP detection tool (NIH and Sanger Institute, UK) in exon 8 and not previously genotyped. All result in amino-acid substitutions, but their phenotypic influences have not been characterized. In the present study, the MAF of rs12135160(G > A) was 0.00042 in 3,768 controls, and consequently, we have no statistical power to detect an association. Both rs28371588(C > A) and DAF-WES^a/b^(G > T) were monomorphic in the case-control collection.

**Table 3:** Genotyping data of non-synonymous SNPs. The numbers of individuals with each genotype in case-control data are indicated. MAF, minor allele frequency calculated from control samples.

SNP	Population	MAF	1/1	1/2	2/2
rs28371588(C > A)	Case	0	3420	0	0
	Control	0	3748	0	0
DAF-WESa/b(T > G)	Case	0	3438	0	0
	Control	0	3738	0	0
rs12135160(G > A)	Case	0.0004	3437	3	0
	Control	0.0004	3765	3	0

## Discussion

In this study, we did not find any evidence for an association between T1D and the *DAF *region in large case-control and family collections using a LD mapping approach. We combined the HapMap II genotyping data and resequencing data, for the selection of tag SNPs. Had we chosen the tag SNPs using only the HapMap II genotyping data, only two tag SNPs (rs2564978 and rs1507765) were required to capture the detected variation within the ~40 kb *DAF *region with a minumum *R*^2 ^of 0.8. However, when the predictive performance of the two tag SNPs were applied to the combined sequence dataset, they no longer captured the variation within the region to the required level since seven of the thirty-six common polymorphisms had an *R*^2 ^below 0.8. The inability of the tag SNPs selected from HapMap II data to tag the combined dataset (minimum *R*^2 ^= 0.35) suggests that for the analysis of localized regions containing candidate genes, as opposed to whole-genome association studies, HapMap II data alone may not provide sufficient information to facilitate a comprehensive LD-mapping approach. In the tag SNP approach, as the causal variant is unknown, we assume that the problem of predicting the causal polymorphism is likely to be no more difficult than that of predicting any other polymorphism[28]. Consequently, the power of the tag SNP approach to detect a causal polymorphism is based upon the minimum *R*^2^[28], assuming that the majority of common polymorphisms in a region are known. In this instance, incomplete knowledge of the common polymorphisms in a region inflated the minimum *R*^2^, providing false confidence in the ability of the tag SNPs to capture the variation within a region, and in the power to detect a causal variant. Our results indicate that for some genes/regions HapMap II data may need to be supplemented by additional resequencing data to allow comprehensive association mapping of common variants.

## Conclusion

We conclude that variation in *DAF *itself is unlikely to have a major effect in T1D in these populations. Analysis of an extended region, surrounding the *DAF *region analysed in this study, showed a cluster of several other genes involved in the complement system, including C4b binding protein (C4bp) and membrane cofactor protein (MCP), both known regulators of complement activation (RCA) genes[11,37,38]. C4bp and MCP restrict complement activation by inhibiting the formation of C3 convertases in the classical pathways like DAF, suggesting that they modulate each other in direct and indirect ways. To clarify the relation of autoimmune disease and complement system, including DAF, further genetic association studies and functional studies on RCA genes are needed. The set of tag SNPs and the LD map for the *DAF *region will be useful for such further studies.

## Methods

### Subjects

The resequencing panel consisted of 32 CEPH individuals; Utah residents with ancestry from Northern and Western Europe collected in 1980 by the Centre d'Etude du Polymorphisme Humain (CEPH).

The 3,523 cases were recruited as part of the Juvenile Diabetes Research Foundation/Wellcome Trust Diabetes and Inflammation Laboratory's United Kingdom Genetic Resource Investigating Diabetes (U.K. GRID) study, which is a joint project between the University of Cambridge Department of Paediatrics and the Department of Medical Genetics at the Cambridge Institute for Medical Research. Most cases were < 16 years of age at the time off collection, all resided in Great Britain, and all were of European descent (self-reported). The 3,817 control samples were obtained from the 1958 British Birth Cohort (1958 BBC), an ongoing follow-up of all person born in Great Britain during one week in 1958 (National Child Development Study)[39]. All cases and control were of white ethnicity.

All families were Caucasian and of European descent, with two parents and at least one affected child. The family collection consisted of 457 multiplex families from the U.K. British Diabetic Association Warren 1 repository[40] and 268 multiplex families from U.S.A. Human Biological Data Interchange[41].

The Cambridge Local Research Ethics Committee gave full ethical approval, and informed consent was obtained for the collection and use of these DNA samples from all subjects.

### *DAF *resequencing

We first annotated the DAF gene locally[42,43] and displayed the annotation through gbrowse[44] within T1DBase[45], using these annotations we resequenced all 11 exons, exon/intron boundaries and up to 3 kb of 3' and 5' flanking sequence of the DAF gene in 32 CEPH individuals, to increase the SNP density across the region. The sequencing reactions were carried out on nested PCR products using Applied Biosystems (ABI) BigDye terminator v3.1 chemistry and the sequences resolved on an ABI3700 DNA Analyser. Polymorphisms were identified using the Staden Package [46] and double-scored by a second operator.

### Statistical analysis

The multilocus test has been described in detail elsewhere[9,28,29,47], briefly, for the case-control data, the multilocus test is essentially Hotellings *T*^2^[48,49], in which we score each diallelic locus as 0, 1 or 2 and compare the mean score vectors between cases and control. In the case of the family data, the multilocus test takes the form of a multilocus TDT[28], in which, for each parent, we calculate a vector whose elements describe transmissions of each of the tag SNPs. If the parent is homozygous at a locus, the corresponding element is scored as zero, otherwise it scored as either +1 or -1 depending on which allele was transmitted. The multilocus test tests the mean of this vector against zero; it is asymptotically distributed as a χ^2 ^with degrees of freedom (df) equal to the number of tag SNPs[28,47].

The program for the selection of tag SNPs[28] and association analysis used here are implemented in the *Stata *statistical system and may be downloaded from our website[50].

### Genotyping

Genotyping was performed using Taqman MGB (Applied Biosytems Inc, Foster City, CA)[51]. All genotyping data were double-scored to minimize error. All genotyping data were in Hardy-Weinberg equilibrium (*P *> 0.05). Genotyping failure rates for all assays in both the family and case-control collection were ≤ 6%.

## Authors' contributions

HT participated in the design of the study, gene annotation, sequencing, genotyping, data analysis and manuscript preparation. CEL participated in the design of the study, genotyping, data analysis and manuscript preparation. JDC participated in data analysis and manuscript preparation. DJS and RB participated in genotyping and sequencing. SN coordinated DNA resources. NMW coordinated data management. LS, BCH, ACL and OB participated in genome informatics. LSW and JAT participated in the conception, design and coordination of the study and participated in manuscript preparation. All authors read and approved the final manuscript.
